# Calcaneum-Achilles Tendon Allograft for Massive Posterosuperior Rotator Cuff Lesion With Bony Deficiency

**DOI:** 10.1016/j.eats.2024.102919

**Published:** 2024-02-03

**Authors:** Alberto Guizzi, Philippe Collin, Jeanni Zbinden, Juan Arturo Hurtado, Arash Amiri, Alexandre Lädermann

**Affiliations:** a Department of Medical and Surgical Specialties, Radiological Sciences, and Public Health, University of Brescia, Brescia, Italy; b CHP Saint-Gregoire, Saint-Grégoire, France; c Clinique Victor Hugo, Paris, France; d American Hospital of Paris, Neuilly-sur-Seine, France; e Division of Orthopaedics and Trauma Surgery, Hôpital de La Tour, Meyrin, Switzerland; f Clínica San Pablo Surco, Lima, Perú; g Clínica San Gabriel, Lima, Perú; h Clínica Providencia, Lima, Perú; i Trauma Research Center, Rajaee (Emtiaz) Trauma Hospital, Shiraz University of Medical Sciences, Shiraz, Iran; j Division of Orthopaedics and Trauma Surgery, Department of Surgery, Geneva University Hospitals, Geneva, Switzerland; k Faculty of Medicine, University of Geneva, Geneva, Switzerland

## Abstract

Dealing with massive and irreparable rotator cuff tears presents intricate challenges. Concerning elder patients, either conservative management or reverse shoulder arthroplasty could be the most appropriate treatment. On the other hand, in younger patients, there is a wide spectrum of solutions, most of them being under evaluation and not completely validated. The complexity increases when a greater tuberosity avulsion occurs at the same time. Regardless of whether surgical fixation is performed, there is a risk for bone resorption, which would result in the posterosuperior cuff's insertion spot loss. In this case, the surgeon is expected to simultaneously manage the bone loss and the tendon tear. The Calcaneum-Achilles Tendon Allograft (CalATA) therefore appears to play an interesting role due to its solid bone-tendon structure. This Technical Note aims to present the CalATA technique, which consists in both tendon and bone deficiency restoration in massive rotator cuff tears with greater tuberosity resorption.

Different approaches for irreparable rotator cuff lesion have been developed. When dealing with elder patients with combined cuff tear arthropathy, the best solution is certainly either conservative treatment or a reverse shoulder arthroplasty.^1^, ^2^, ^3^ In the case of younger patients, however, prosthetic replacement is no longer the first-choice option due to implant survival rates and long-term complications.^4^, ^5^, ^6^ Traditionally, different alternatives to prosthetics in younger patients include partial repair,^7^ tendon transfers,^8^ or more controversial options such as superior capsular reconstruction^9^^,^^10^ and subacromial spacers.^11^

In traumatic massive injuries, a greater tuberosity avulsion may be involved. Depending on the type of fracture, it could be treated conservatively or surgically, but in both cases, bone resorption of the detached fragment may occur.^12^ This way, the footprint of the posterosuperior rotator cuff would be lost, classified as A1 superior rotator cuff lesions.^13^ Such situations are challenging to manage when bone support is missing, especially in young patients^14^: theoretically, the surgeon is required to restore the bone stock and provide a robust rotator cuff repair at the same time. The Calcaneum-Achilles Tendon Allograft (CalATA) technique was described as an alternative treatment to joint replacement in a young patient with greater tuberosity and rotator cuff deficiency.^15^ Ho et al.^16^ demonstrated good clinical results, tendon healing, and bone incorporation in 5 patients at minimum 2 years of follow-up.

The purpose of this Technical Note is to describe the CalATA technique, which involves the use of a calcaneus bone graft along with its attached Achilles tendon. This technique is used to restore both the bone and tendon components simultaneously in cases of massive rotator cuff tears with greater tuberosity deficiency.

## Technique

### Preoperative Decision-Making

**Fig 1:**
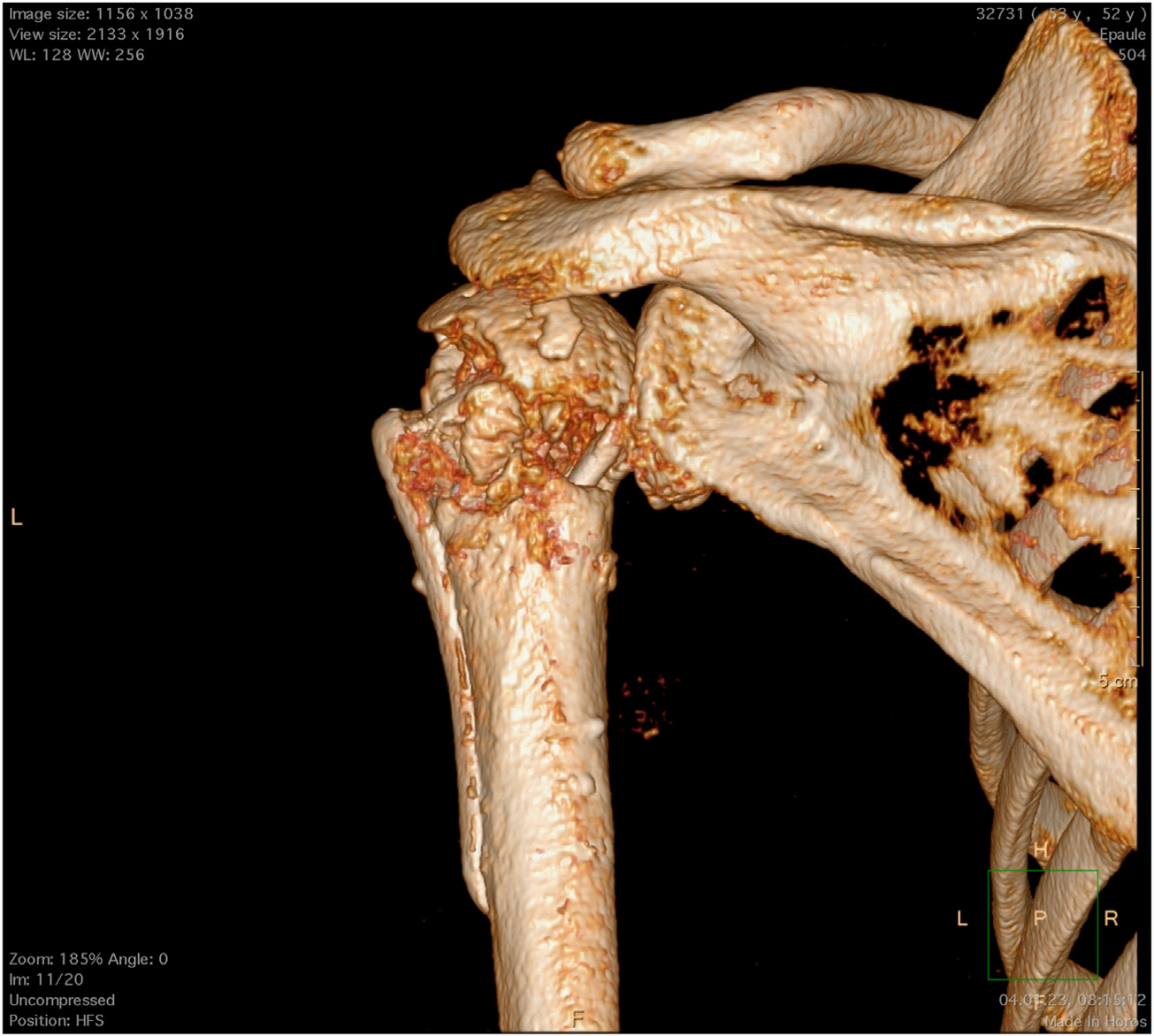
Three-dimensional computed tomography scan reconstruction of a left shoulder from a posterior point of view. A greater tuberosity deficiency can be noted.

The rotator cuff tear’s diagnosis is typically made through patient history, clinical examination, and imaging (Fig 1). Standard imaging evaluation consists of the following:

- Radiographic evaluation (true anteroposterior and axillary views) to exclude arthritic changes
- Magnetic resonance imaging (MRI) to assess tear characteristics, muscle fatty infiltration, and muscle atrophy
- Computed tomography could be helpful to better quantify the bone defect when greater tuberosity bone resorption is shown at radiographic evaluation and MRI, or in case of hardware to limit artifacts.

Indications, contraindications, and surgical pearls and pitfalls for the CalATA technique are described in Tables 1 and 2, respectively.

**Table 1:** Possible Indications and Contraindications for the Calcaneum-Achilles Tendon Allograft Technique

Indications	Contraindications
Massive reparable or irreparable posterosuperior rotator cuff lesion associated with greater tuberosity bone loss	Associated irreparable subscapularis lesion
	Pseudoparalytic shoulder with anterosuperior escape and/or acromiohumeral distance <7 mm
	Glenohumeral osteoarthritis or humeral head avascular necrosis
	Grade 3-4 fatty infiltration of the posterosuperior rotator cuff according to Goutallier et al.^30^

**Table 2:** Surgical Pearls and Pitfalls for the Calcaneum-Achilles Tendon Allograft Technique

Pearls	Pitfalls
Maintain previous hardware if doubt on bone healing	Insufficient tuberosity bone surface debridement would prevent bone-to-bone healing Risk of calcaneus bone block fracture during fixation and remodeling
Transdeltoid approach for better management of the posterosuperior rotator cuff and residual avulsed bony fragments	Excessive tendon graft length resulting in not enough tension
Use Gerber subacromion retractor to expose residual rotator cuff	Tendon too thinned out and therefore too weak
Prepare repair by passing numerous sutures in the residual cuff using a suture passer before CalATA implantation	Excessive small size of bone graft with risk of fracture while screwing the malleolar screw
Use intraoperative fluoroscopic guidance to check the proper position of the malleolar screw	Malleolar screw too long with intra-articular perforation risk

CalATA, Calcaneum-Achilles Tendon Allograft.

### Patient Setup, Native Cuff, and Bone Remnant Exposure

The patient is placed in a beach-chair position under general anesthesia with an interscalene nerve block. Depending on whether the patient had a previous surgery or not, the skin incision might be overlapped to the previous one. Otherwise, an anterosuperior incision is performed to complete a deltoid split approach, so that the posterior rotator cuff can be properly exposed (Video 1).

**Fig 2:**
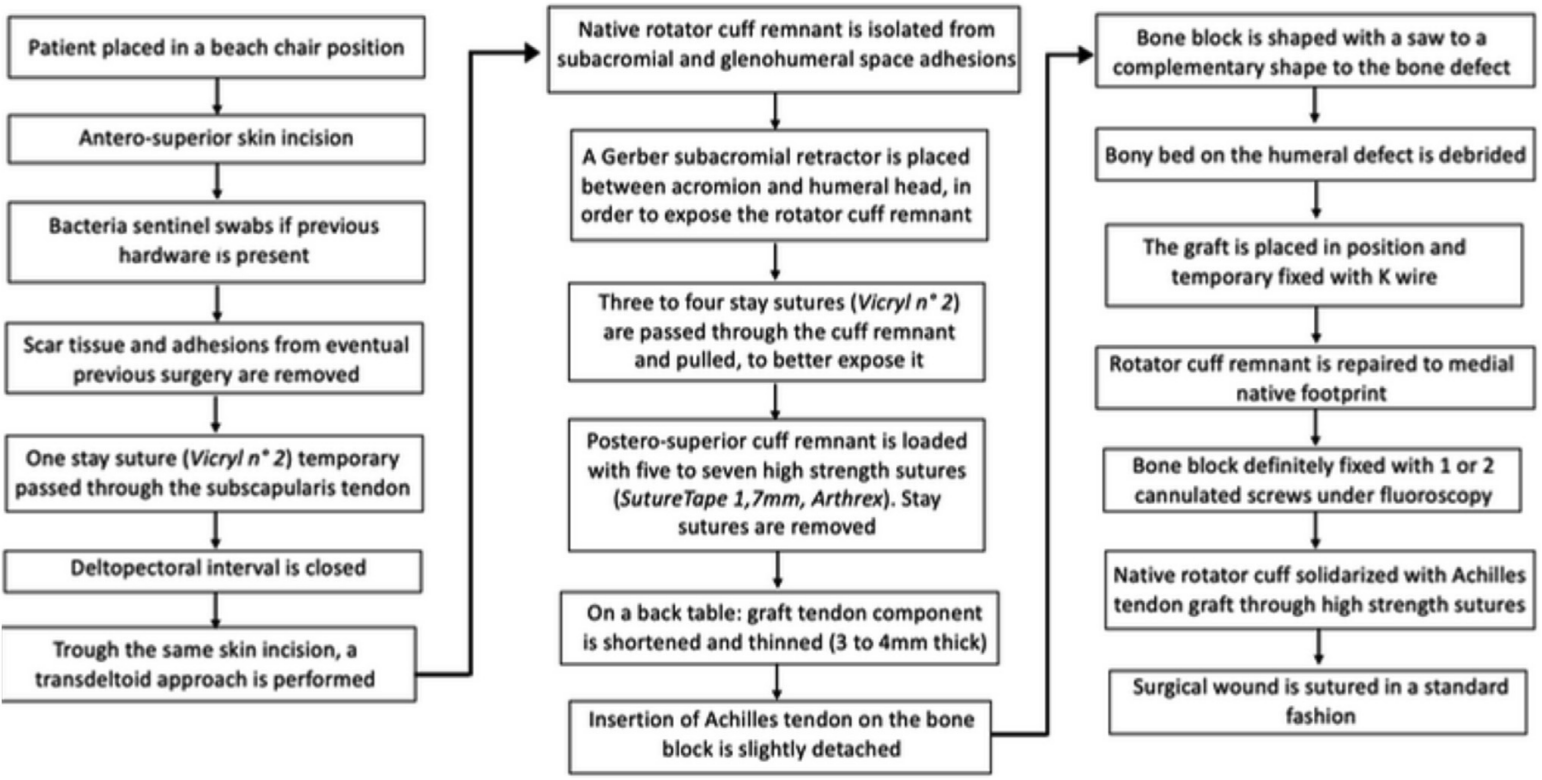
Outline of the Calcaneum-Achilles Tendon Allograft step-by-step procedure.

A step-by-step description of this procedure is shown in Figure 2.

**Fig 3:**
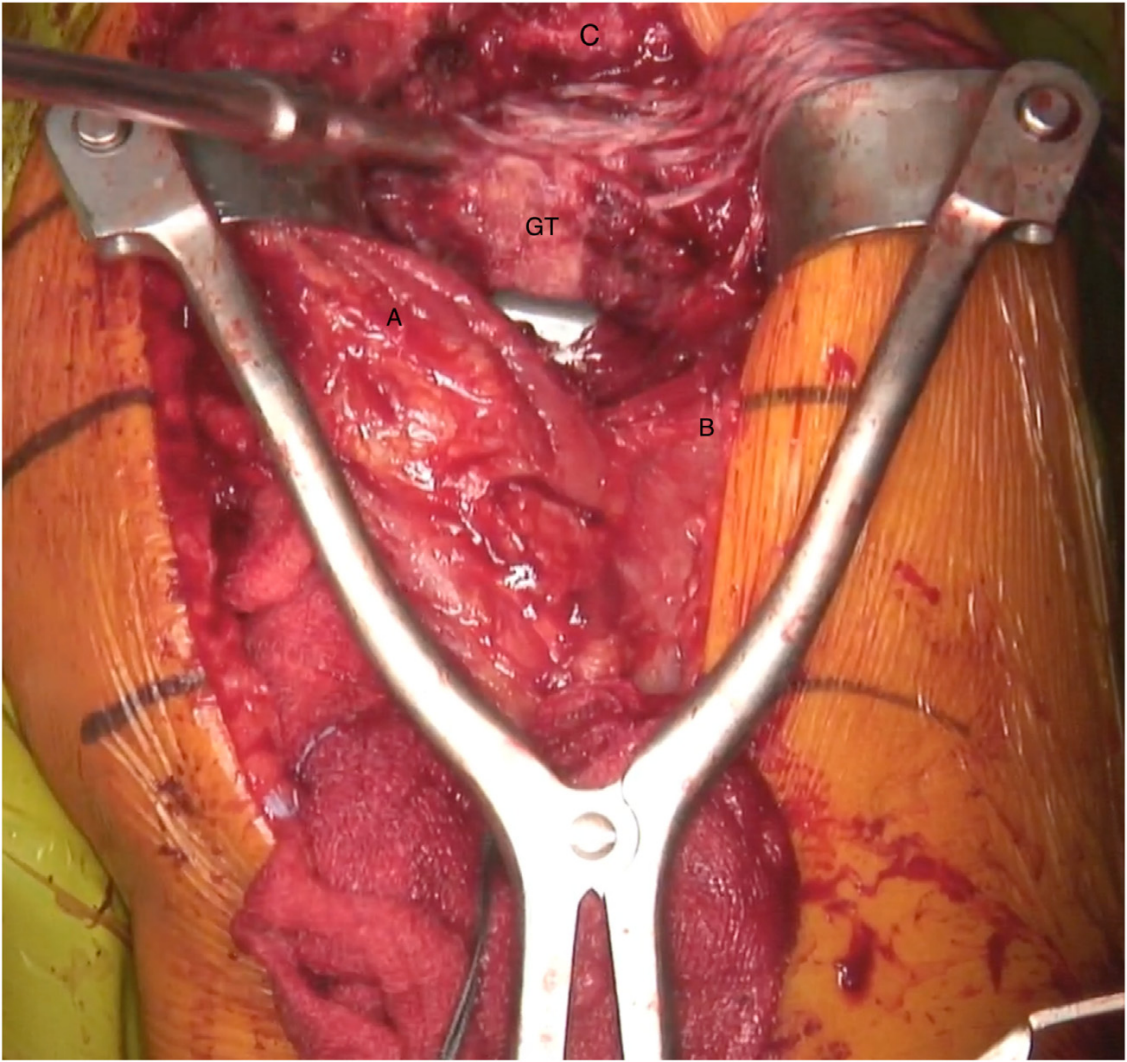
Lateral transdeltoid view of a left shoulder. The proximal humeral head defect is exposed and debrided as well, until a viable bony bed is reached, to better identify the real shape of the bone loss. (A, deltoid muscle anterior side; B, deltoid muscle posterior side; C, acromion; GT, greater tuberosity deficiency.)

**Fig 4:**
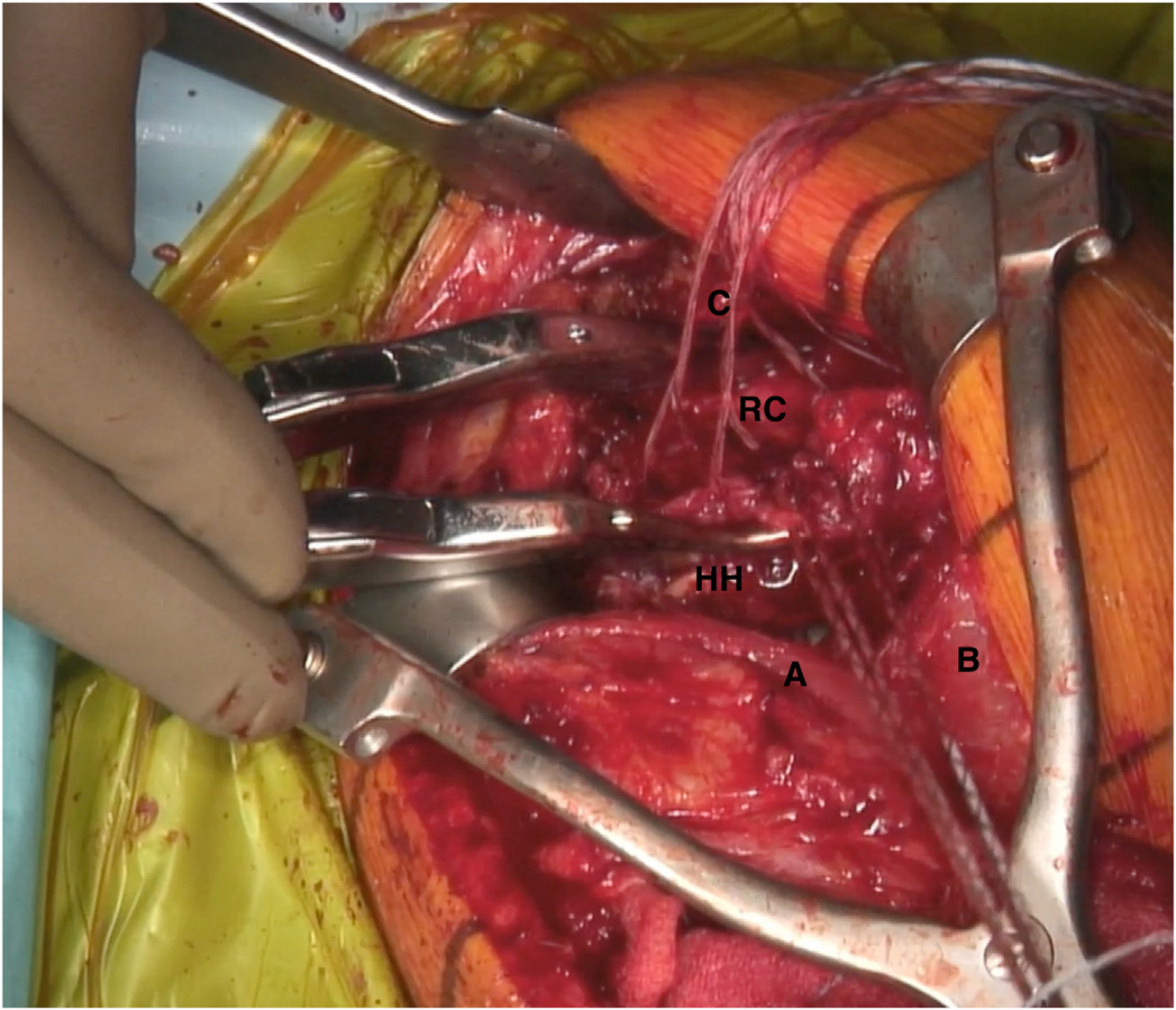
Lateral transdeltoid view of a left shoulder. A Gerber subacromion retractor is placed, so that the cuff remnant is well exposed. The posterosuperior cuff remnant is prepared with 5 to 7 high-strength sutures using either suture passer or small needles. If the remnant tissue is not easily accessible, 3 to 4 stay sutures can be temporarily passed through the cuff to pull and optimally expose it. (A, deltoid muscle anterior side; B, deltoid muscle posterior side; C, acromion; HH, humeral head; RC, native rotator cuff remnant.)

Whether previously installed hardware is present, we suggest performing sentinel swabs to rule out eventual germ proliferation and active infective processes. In case of previous proximal humeral fracture and according to the elapsed time since the fracture’ synthesis, hardware removal is suggested thereafter. The remnant rotator cuff tendon is identified, debrided, and preserved. The proximal humeral head defect is then exposed and debrided as well, until a viable bony bed is reached, to better identify the real shape of the bone loss (Fig 3). A Gerber subacromion retractor is placed, so that the cuff remnant is well exposed (Fig 4 and Video 1). The posterosuperior cuff remnant is prepared with 5 to 7 high-strength sutures (SutureTape 1.7 mm; Arthrex) using either a suture passer or small needles (Fig 3). If the remnant tissue is not easily accessible, 3 to 4 stay sutures (Vicryl No. 2) can be temporarily passed through the cuff to pull and optimally expose it.

### Graft Preparation

**Fig 5:**
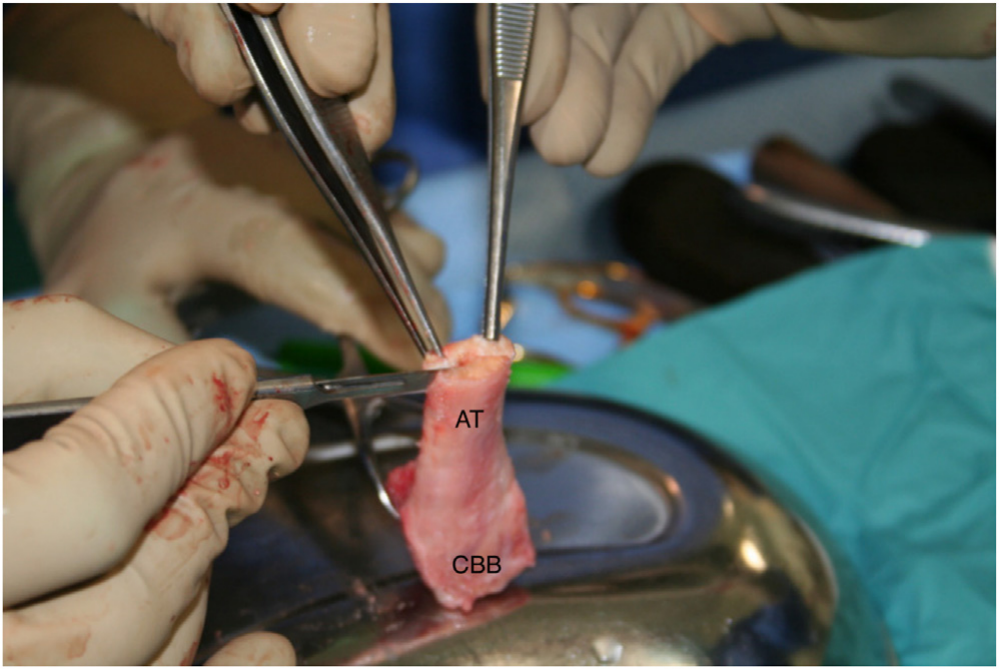
Fresh-frozen, gamma-irradiated CalATA is thawed and prepared. The Achilles tendon is thinned using a cold blade scalpel until a reasonable thickness of around 3 to 4 mm. (AT, Achilles tendon; CBB, calcaneus bone block.)

**Fig 6:**
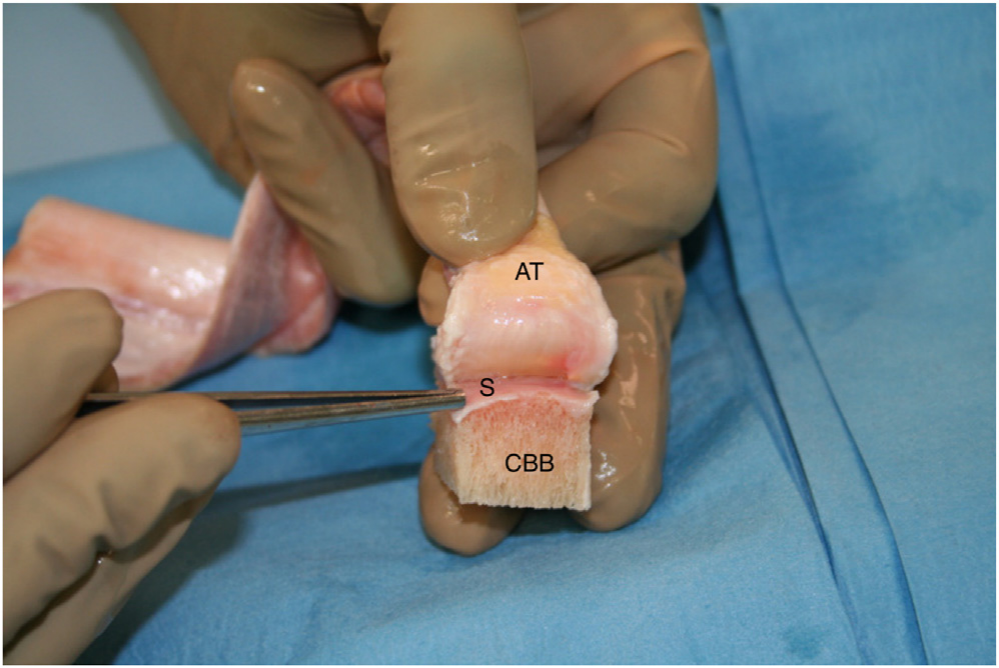
The Achilles tendon is partially lifted off the bone block to create a shelf of bone adjacent to the tendon. This is to allow for improved rotator cuff tendon ingrowth after the native rotator cuff repair. (AT, Achilles tendon; CBB, calcaneus bone block; S, shelf.)

**Fig 7:**
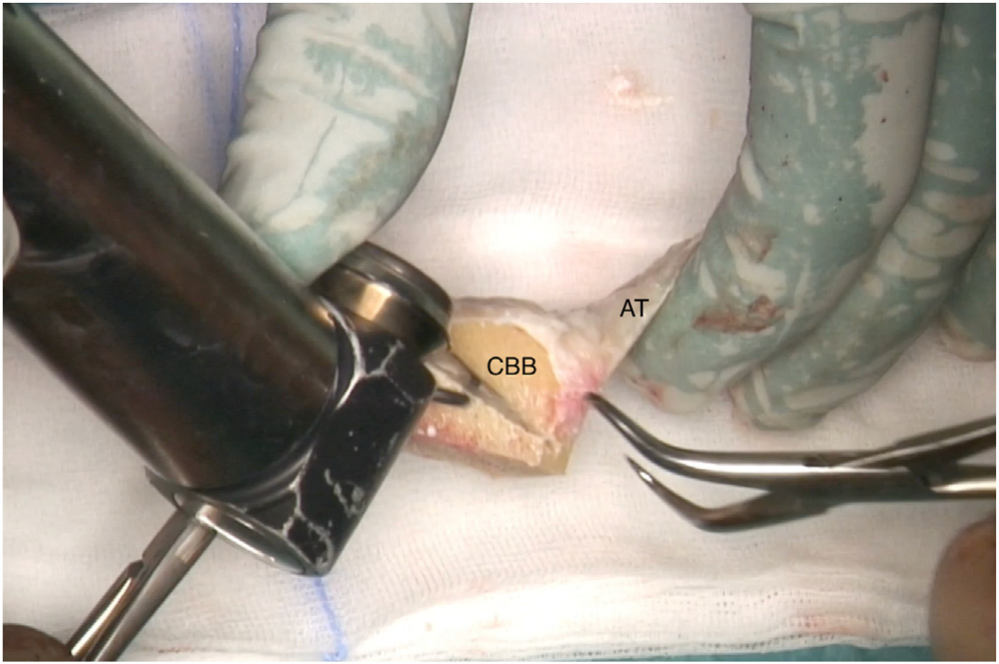
Depending on the size of the greater tuberosity defect, the calcaneus is shaped using a saw to obtain a graft that can fit as much as possible into the bone humeral defect. (AT, Achilles tendon; CBB, calcaneus bone block.)

In the meantime, a fresh-frozen, gamma-irradiated CalATA is thawed in tempered saline solution for 15 minutes. At first, depending on graft characteristics, the Achilles tendon can be thinned using a cold blade scalpel until a reasonable thickness of around 3 to 4 mm (Fig 5). Using the same blade, the insertion of the Achilles tendon to the calcaneus block is slightly detached to create a shelf. This will allow the native rotator cuff remnant ingrowth after repair (Fig 6). Thereafter, the bony block is prepared. Depending on the size of the greater tuberosity defect, the calcaneus is shaped using a saw, to obtain a graft that can fit as much as possible into the bone humeral defect (Fig 7 and Video 1).

### Graft Fixation and Rotator Cuff Repair

**Fig 8:**
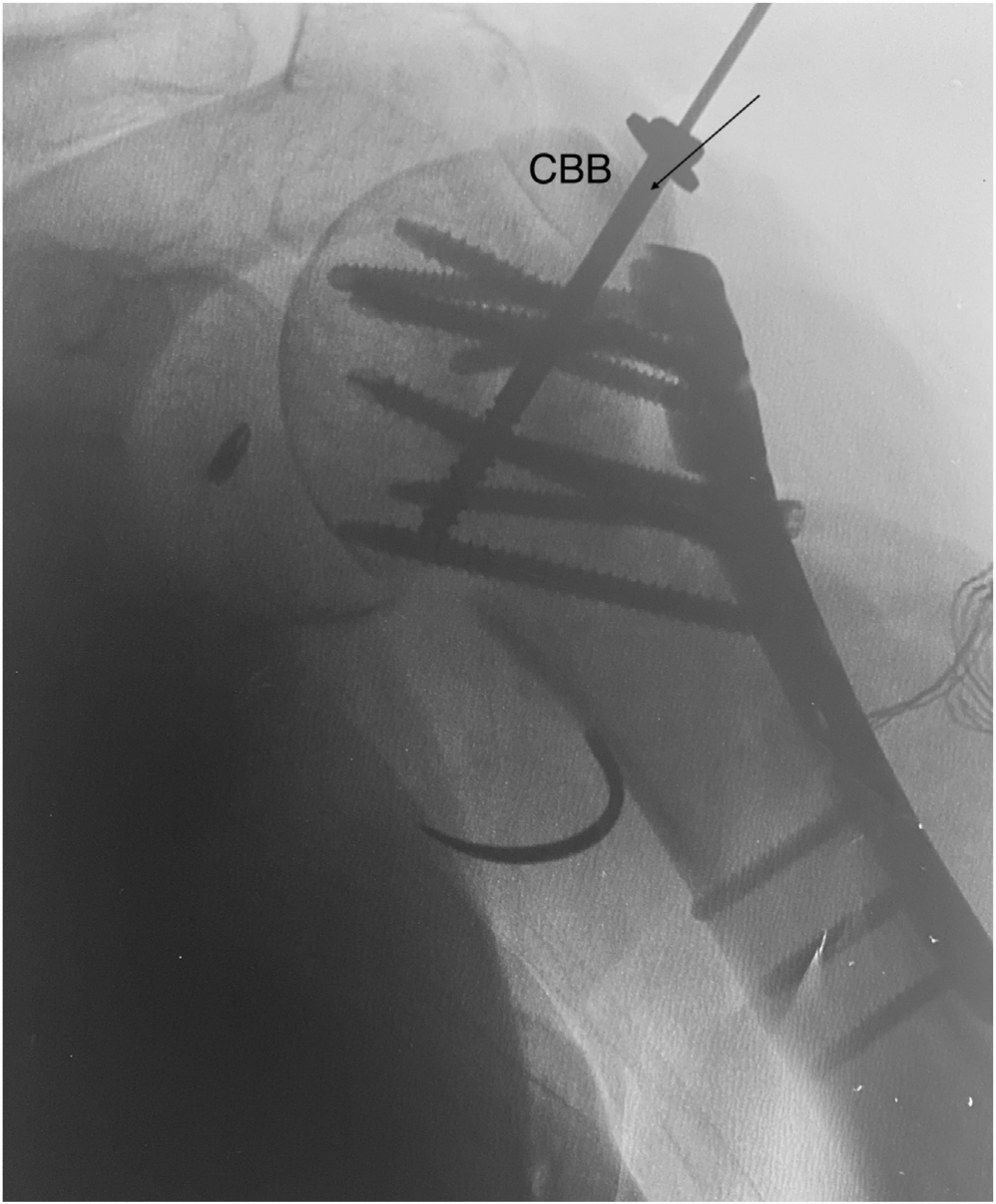
Intraoperative fluoroscopic anteroposterior image of a left shoulder. Restoration of bone stock and good fixation of the calcaneum graft are confirmed. The plate fixing the initial fracture has been left in place due to insufficient consolidation. Arrow indicates malleolar screw fixing the bone block. (CBB, calcaneus bone block.)

**Fig 9:**
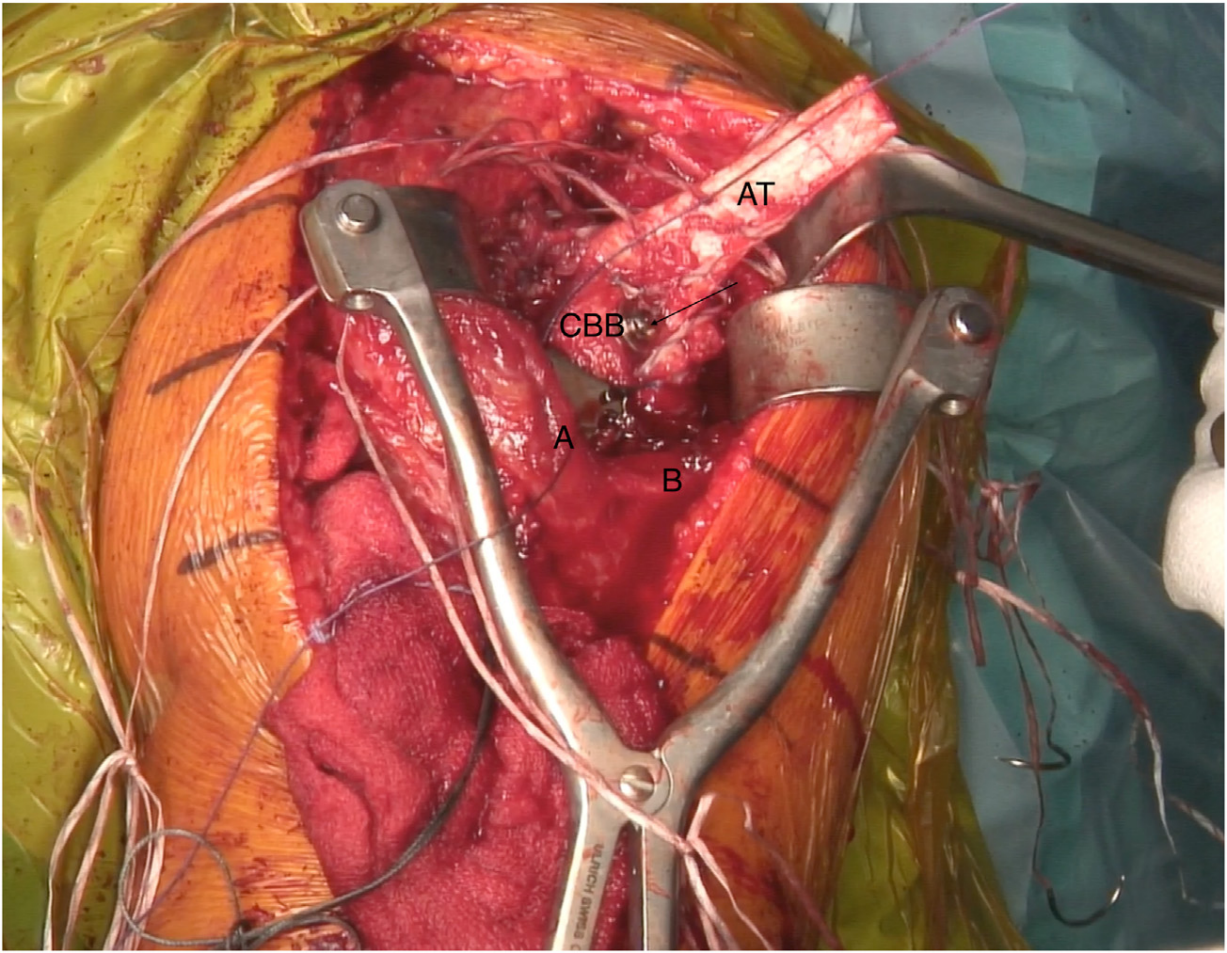
Lateral transdeltoid view of a left shoulder. The Calcaneum-Achilles Tendon Allograft is fixed to the proximal humerus with 1 cannulated screw. The tendinous part of the graft is flipped laterally to better expose the native rotator that is repaired. Arrow indicates malleolar screw fixing the bone block. (A, deltoid muscle anterior side; AT, Achilles tendon; B, deltoid muscle posterior side; CBB, calcaneus bone block.)

**Fig 10:**
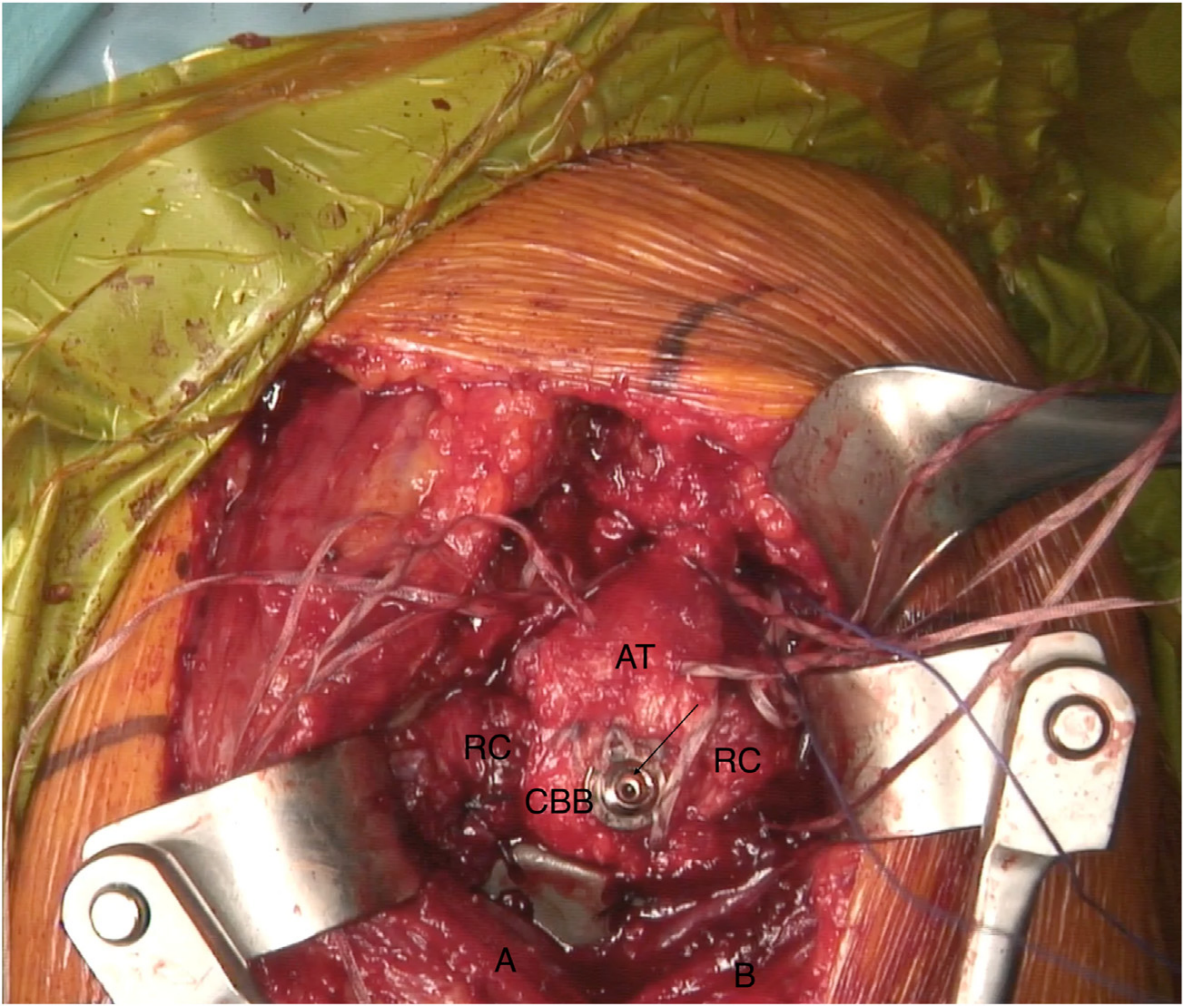
Lateral transdeltoid view of a left shoulder. The Achilles tendon is sewn with high-strength sutures onto the remnant cuff to reinforce the repair. Arrow indicates malleolar screw fixing the bone block. (A, deltoid muscle anterior side; AT, Achilles tendon; B, deltoid muscle posterior side; CBB, calcaneus bone block; RC, native rotator cuff remnant.)

Depending on the size of the final graft acquired, this is fixed onto the proximal humerus defect through the bony portion with either one or two 4-mm malleolar screws (Asnis cannulated screws, 4 mm diameter; Stryker) under fluoroscopic control (Fig 8). Once the bony part is properly fixed, the tendinous part of the graft is flipped laterally to better expose the medial native bone remnant, on which the native rotator cuff is repaired using modified Mason-Allen knots. The goal is then to reinforce the original rotator cuff by making it solid with the Achilles tendon graft (Fig 9). The latter is therefore shortened to the appropriate length and sewn onto the remnant cuff using high-strength sutures (SutureTape 1.7 mm; Arthrex) (Fig 10 and Video 1).

### Postoperative Rehabilitation

Postoperatively, the arm is immobilized in an abduction pillow in neutral rotation for 6 weeks. Afterward, the sling is removed, and passive mobilization is practiced until week 12. Then, active mobilization progressively begins until the patient returns to full activity levels.

## Discussion

In this Technical Note, we presented an augmentation method with CalATA as a solution for the treatment of massive rotator cuff injuries with associated greater tuberosity bone insufficiency.

Besides the footprint of the 3 rotator cuff tendons, the absence or displacement of the greater tuberosity also generates an alteration of the regular humeral head shape. This way, the force vector of the deltoid muscle is medialized, and consequently, the so-called deltoid-wrapping effect is lost.^17^ The “deltoid-wrapping” concept is gaining recognition in shoulder arthroplasty. It suggests that a decrease of deltoid wrapping angle leads to a reduction in deltoid tension.^17^ Even if there is currently no specific literature available analyzing the deltoid-wrapping effect in case of native humeral head bone loss, it makes sense to apply the same principle. When the greater tuberosity contour is lost, the deltoid-wrapping effect over the native humeral head is compromised. As a result, there is diminished deltoid tension, leading to decreased function. This further emphasizes the importance for the surgeon to consider repairing both the bone and tendon components.

Achilles tendon alone was already known in literature for being useful and successful in the treatment of other ligamentous and tendinous reconstructions throughout the body.^18^, ^19^, ^20^ Its use into the shoulder was introduced by Mease et al.,^21^ who proposed a superior capsular reconstruction technique with CalATA. The latter was already described for its usefulness in knee surgery^22^ but was first introduced into the shoulder field in the case report by Lädermann et al.^15^ and consequently overviewed by other authors.^16^^,^^23^^,^^24^

The aforementioned case report^15^ was about a 44-year-old woman with a massive, nonretracted rotator cuff lesion combined with osteonecrosis and complete resorption of the greater tuberosity. Excellent clinical-radiologic results were reported after CalATA implantation. The positive outcomes were further reinforced in a case series,^16^ which involved 5 patients diagnosed with reparable massive type D lesions accompanied by greater tuberosity insufficiency.^13^^,^^25^ These patients underwent the CalATA procedure. Clinical and radiologic results were satisfactory for 4 of the 5 patients. The 1 unsuccessful case clinically manifested with an avascular necrosis progression through the entire humeral head and ended with a prosthetic replacement 1 year after the previous surgery.

However, these satisfactory results run against the findings by Kholinne et al.^24^ In a case series of 6 patients, the authors studied the validity of CalATA for superior capsular reconstruction in the management of irreparable massive cuff injuries. The rationale behind the study is based on animal studies^26^^,^^27^ that showed that the healing process at the bone-to-bone interface is more favorable than at the tendon-to-bone interface. Therefore, instead of using the Achilles tendon alone for superior capsule reconstruction, they proposed to preserve the calcaneal bone fragment and implant the entire structure as a unified entity, facilitating bone-to-bone healing. However, the clinical results were highly unfavorable, with failure observed in 83% of cases, in contrast to the moderate success reported in the earlier mentioned studies.^15^^,^^16^ This notable disparity can potentially be attributed to the differences in patient injuries between the case series,^16^ which involved repairable massive injuries, and the study by Kholinne et al.,^24^ which dealt with irreparable injuries. Consequently, in the first scenario, the Achilles tendon served as an augment to the original cuff repair with a wide zone of contact between the allograft and the superior cuff, whereas in the second one, it functioned as a reconstructive element for the superior capsule with a tiny zone of contact between the bones and the allograft.

The CalATA shows its desirability since by having a single compact structure, both cuff and bone defects can be simultaneously managed. In addition, the robustness of the Achilles tendon and the exceptional malleability of the calcaneal bone component make CalATA extremely attractive. Clearly, the allograft use carries some advantages and disadvantages. Advantages include that it has no donor site morbidity and is less time-consuming.^28^ On the other hand, it has less biological properties when sterilized with certain techniques, which results in a lower healing rate.^28^^,^^29^ Consequently, clinical evidence about the efficacy of the CalATA technique is surely needed, but it seems that the underlying anatomic and biomechanical principles represent a solid starting point for further studies.

## Conclusions

CalATA appears to play an interesting role in the management of massive rotator cuff lesions when associated with greater tuberosity bony deficiency. Due to its solid bone-tendon structure, both bone and tendon defects can be managed at the same time. Moreover, construct solidity is further increased since the bone-to-bone interface heals better than the tendon-to-bone interface.

## Disclosures

The authors report the following potential conflicts of interest or sources of funding: Supported by FORE (Foundation for Research and Teaching in Orthopedics, Sports Medicine, Trauma, and Imaging in the Musculoskeletal System), Grant 2023-69. P.C. receives royalties from and is a paid consultant and a paid speaker for Stryker and Enovis. He is the co-founder of Med4Cast and Follow Health. He is on the board of SESEC and IBSES. A.L. is a paid consultant for Arthrex, Stryker, Medacta, and Enovis; has received royalties from Stryker and Medacta; is the (co)founder of FORE, Med4Cast, and BeeMed; owns stock options in Medacta and Follow Health; and is on the board of the French Arthroscopic Society. All other authors (A.G., J.Z., J.A.H., A.A.) declare that they have no known competing financial interests or personal relationships that could have appeared to influence the work reported in this paper*.* Full ICMJE author disclosure forms are available for this article online, as supplementary material.

## Supplementary Data

ICMJE author disclosure forms

Video 1 This video describes the Calcaneum-Achilles Tendon Allograft (CalATA) technique for massive posterosuperior rotator cuff lesions with bony deficiency. Computed tomography scan and magnetic resonance imaging respectively show bony deficiency on the greater tuberosity and rotator cuff tear after proximal humeral fracture’s fixation. The patient is placed in a beach-chair position. An anterosuperior skin incision is performed unless there is a previous scar to resume (like in this case on video). Whether previously installed hardware is present, bacteria sentinel swabs are recommended to rule out eventual germ proliferation. Tissues are freed from adhesions from any previous surgical treatment and scar tissue is removed. One stay suture is temporary passed through the subscapularis tendon using a Vicryl No. 2. In this case, the deltopectoral interval is closed to move to the transdeltoid approach, so that the greater tuberosity can be properly exposed. Native rotator cuff remnant is isolated and adhesions from both the subacromial and glenohumeral space are removed. A Gerber subacromion retractor is placed, so that the cuff remnant can be well exposed. The posterosuperior cuff remnant is prepared with 5 to 7 high-strength sutures using a suture passer. When the native tissue is not easily accessible, 3 to 4 stay sutures can be temporarily passed through the cuff to pull and optimally expose it (like in this case). Stay sutures are removed. Then, on a back table, the CalATA is prepared. Depending on graft features, the tendon component is shortened and thinned until a proper length and 3 to 4 mm of thickness. The insertion of the Achilles tendon to the calcaneus block is slightly detached to create a shelf. This will allow the native cuff ingrowth after repair. The bony block is prepared with a saw to obtain a proper shape that can fit as best as possible in the humeral bone defect. Bony bed on the greater tuberosity is crucial in order not to obstruct bone-to-bone healing. The graft is placed and temporarily fixed with a K-wire in the correct position. Rotator cuff remnant is repaired to the medial native bone remnant using modified Mason-Allen knots through the plate. Depending on the size of the final graft, this is finally fixed with one or two 4-mm malleolar screws under fluoroscopic guidance. Native rotator cuff is then reinforced and solidarized with the Achilles tendon graft through high-strength sutures. The surgical wound is then sutured in a standard fashion.
